# Aerobic Glycolysis: Beyond Proliferation

**DOI:** 10.3389/fimmu.2015.00227

**Published:** 2015-05-15

**Authors:** William Jones, Katiuscia Bianchi

**Affiliations:** ^1^Centre for Molecular Oncology, Barts Cancer Institute, Queen Mary University of London, London, UK

**Keywords:** aerobic glycolysis, non-malignant cells, cellular signalling, proliferation, immunometabolism

## Abstract

Aerobic glycolysis has been generally associated with cancer cell proliferation, but fascinating and novel data show that it is also coupled to a series of further cellular functions. In this *Mini Review*, we will discuss some recent findings to illustrate newly defined roles for this process, in particular in non-malignant cells, supporting the idea that metabolism can be considered as an integral part of cellular signaling. Consequently, metabolism should be regarded as a plastic and highly dynamic determinant of a wide range of cellular specific functions.

The term aerobic glycolyis was coined by Otto Warburg at the beginning of the nineteenth century to explain the unconventional metabolism exhibited by tumor cells. Warburg noticed that malignant cells prefer to convert glucose to lactate even in the presence of oxygen, in contrast to the metabolism of healthy/differentiated cells, where glucose is usually converted into pyruvate and only converted to lactate in the absence of oxygen. The conversion of glucose to lactate in anaerobic conditions (absence of oxygen) was already known as anaerobic glycolysis and thus he defined the metabolism of cancer cells as aerobic glycolysis, to underline that the fate of glucose is not determined by the lack of oxygen (1). The fact that cancer cell metabolism is different from that of normal cells attracted (and still does attract) a lot of interest, since this feature can potentially facilitate selective therapeutic targeting of tumor cells. Currently, we also come across a growing interest in the metabolism of non-malignant cells, particularly of those involved in the immune response. Intriguingly, recent findings in this new field of immunometabolism showed that aerobic glycolysis can be a metabolic choice of normal cells and that its function is not limited to supporting proliferation. It appears that cells can modulate their metabolism to adapt to different energy requirements and signaling events in physiological situations (see Figure 1). Thus, changes in cellular metabolism are not only restricted to nutrient deprivation or pathological conditions. These studies also demonstrate that, despite our fairly detailed knowledge of the map of cellular metabolic reactions, little is known about the link between metabolism and non-metabolic cellular functions. Thus, we can pose intriguing questions: can cellular metabolism determine seemingly unrelated cellular functions and do cells use the modulation of their metabolism to accomplish and regulate these functions?

**Figure 1:**
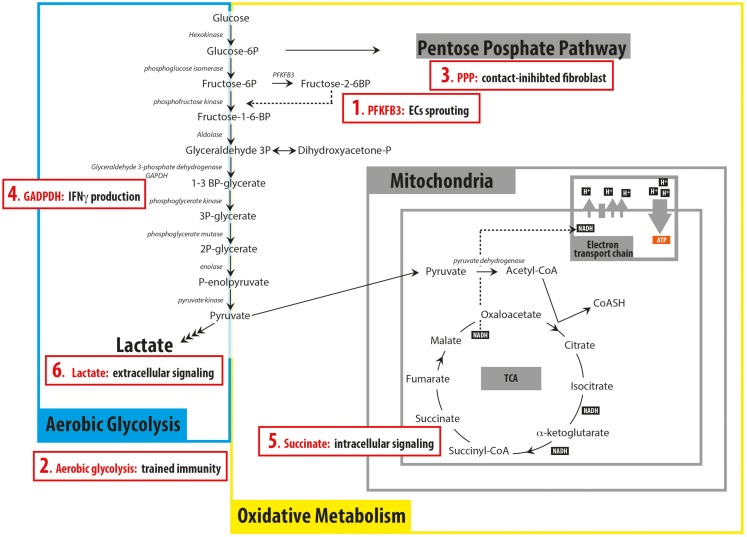
**Schematic representation of the principal pathways involved in glucose catabolism summarizing recent findings discussed in the *Mini Review* (red boxes)**. (1) 6-phosphofructo-2-kinase/fructose-2, 6-biphosphatase 3 (PFKFB3) has a key role in regulating endothelial cells (ECs)sprouting; (2) switching to aerobic glycolysis is important for trained immunity in monocytes; (3) contact-inhibited fibroblasts rely on the pentose phosphate pathway (PPP) to produce NADPH; (4) GAPDH can function as an mRNA binding protein when not engaged in glycolysis, preventing the production of IFNγ; (5) succinate can act as intracellular signaling molecule, being involved in the production of IL-1β in BMDM; (6) Lactate secreted by cancer cells can act as extracellular signaling molecule, inducing the differentiation of macrophages into tumor-associated macrophages.

## A Role for Aerobic Glycolysis in Vessel Sprouting

The process of vessel sprouting requires endothelial cells (ECs) to differentiate into different cell types and act in a synchronized manner. While ECs differentiated into “stalk” cells proliferate and sustain vessel sprouting, the EC differentiated into the “tip” cell is required at the forefront and has a migratory phenotype. Intriguingly, from a metabolic point of view, ECs are more similar to cancer cells than to other normal differentiated cells. ECs are highly glycolytic (it has been calculated that up to 85% of ATP in ECs is produced by glycolysis) despite the fact that they are in immediate contact with oxygen present in the blood (2). In agreement with this observation, it has been reported that the amount of lactate secreted by ECs does not change upon a reduction of oxygen pressure, highlighting the fact that aerobic glycolysis is the preferred metabolism of ECs (3). While vessel sprouting was primarily thought to be regulated by Notch signaling (4), recently, cellular metabolism has also been shown to play a major role in the process. In particular, an important function for 6-phosphofructo-2-kinase/fructose-2,6-biphosphatase 3 (PFKFB3) has been proposed (2). PFKFB3 is responsible for the formation of fructose 2,6 bisphosphate, a key regulator of glycolysis since it acts as the allosteric activator of phosphofructokinase 1 (PFK1), the rate limiting enzyme that converts fructose 6-phosphate to fructose 1-6 bisphosphate. Modulation of the level of expression of PFKFB3 by knockdown or overexpression changes the behavior of ECs growing in spheroids. Knockdown of the enzyme inhibits sprouting, while its overexpression leads to increased glycolysis, together with more and longer sprouts. Importantly, in mosaic spheroids formed of wild-type and PFKFB3 knock-down cells, predominantly wild-type cells form the tips. This preferential selection of wild-type cells is not due to the reduced proliferation rate caused by PFKFB3 silencing, since even when cell proliferation is inhibited by MitoC cells distribute with a similar pattern as before. Moreover, this distribution is maintained up to an initial ratio of wild-type/PFKFB3 knock-down of 9:1, indicating that presence of PFKFB3 is crucial to develop the tip cell phenotype. Importantly, PFKFB3 depletion also abrogates the effect of other factors promoting the tip phenotype, such as inhibition of Notch signaling. Mechanistically, PFKFB3 (and other glycolytic enzymes) has been shown to interact with actin and localize at lamellopodia, possibly to provide fast and local ATP production, where it is required to promote cell motility. Altogether, the role of PFKFB3 and glycolysis in promoting the phenotype of ECs is a clear example of how metabolism affects cellular phenotype and function beyond its role in proliferation. PFKFB3 seems to be also a promising therapeutic target. The small molecule inhibitor of PFKFB3 3-(3-pyridinyl)-1-(4-pyridinyl)-2-propen-1-one (3PO) reduces the glycolytic flux and vessel formation both *in vitro* (EC spheroids) and *in vivo* (in zebrafish and mouse retina) (5). Intriguingly, it has been recently shown that ECs have another rather unique metabolic characteristic, in that they rely on fatty acid oxidation for the production of nucleotides (6). However, this aspect of ECs metabolism is in fact linked to supporting proliferation, and thus lies outside the topic of this mini-review.

## A Role for Aerobic Glycolysis in Trained Immunity

The fact that monocytes and polymorphonuclear leukocytes from peritoneal exudates rely on glycolysis as their main energy source was described in 1963 (7). However, a crucial role for glycolysis has also been recently demonstrated for the development of “trained immunity” (8). Trained immunity can be defined as “memory” of the innate immune response and is responsible for mounting a non-specific “adaptive” response, challenging the concept that the innate immune response cannot have a long-term memory. Evidence for this process has been collected in various *in vitro* and *in vivo* models. For example, monocytes exposed to β-glucan (a wall component of *Candida albicans*) *in vitro* respond to lipopolysaccharide (LPS) with greater amplitude 7 days after “priming,” as the result of the activation of a pathway involving Akt/mTOR and HIF1α. Monocytes exposed to β-glucan for 24 h switch to aerobic glycolysis, and via epigenetic modification sustain this metabolic choice for at least 7 days after having being “primed.” Primed monocytes are more responsive to LPS treatment as measured by the increased amount of TNFα that they secrete when compared to un-primed cells. Importantly, the switch to aerobic glycolysis during the first 24 h of priming is an absolute requirement for monocytes to undergo “training,” together with epigenetic modifications and activation of the Akt/mTOR/HIF1α pathways. The amount of lactate secreted during β-glucan priming is not affected by treatment with methylthioadenosine (MTA), an inhibitor of methyltransferase, or ITF2375, a histone deacetylase inhibitor, but training is abolished by these drugs. Thus, aerobic glycolysis is not a consequence of epigenetic modifications rather both represent a crucial mechanism contributing to trained immunity. Mechanistically, it is not clear why glycolysis favors the adaptive monocyte response. It has been suggested that it can be a swift way to produce energy during infection, where a quick and robust response is required (8). An intriguing hypothesis is that metabolism could affect the epigenetic status, as demonstrated in other instances where DNA modification depends on metabolites acting as cofactors or substrates (9). Along these lines, the ratio between α-ketoglutarate (α-KG) and succinate regulates histone demethylases maintaining pluripotency of embryonic stem cells (10).

## High Level of Glucose Uptake is Not Equal to Proliferation

The observation that aerobic glycolysis was the preferred metabolic pathway of highly proliferative cancer cells was initially difficult to explain from an energetic point of view. Indeed, via glycolysis/OXPHOS cells generate 36 molecules of ATP for each molecule of glucose, while via aerobic glycolysis only 4 molecules of ATP are produced. This apparent paradox was resolved however, by considering the metabolic requirements for proliferation beyond ATP, including nucleotide synthesis linked to the pentose phosphate pathway (PPP), generation of amino acids for protein synthesis, and production of lipids for membrane formation. Importantly, most of these biosynthetic pathways branch out from glycolysis, explaining why this process is so crucial for cellular proliferation (11). It would therefore be logical to assume that non-proliferating or quiescent cells would require less glycolytic activity but, surprisingly, this does not always seem to be the case. Indeed, fibroblasts that are pushed into a quiescent status because of contact inhibition have a very similar glucose uptake rate to proliferating fibroblasts (12). An in depth analysis of the metabolism of quiescent vs. proliferative fibroblasts highlighted some unexpected findings. Proliferating fibroblasts rely on the PPP to generate ribose for the synthesis of nucleotides and to produce NADPH, their TCA cycle is interrupted between citrate and α-KG and they use glutamine as an anaplerotic substrate. Quiescent, contact-inhibited fibroblasts also maintain an active PPP to produce NADPH but, since they do not require nucleic acid synthesis, the carbon skeletons are recycled back to glycolysis. Their TCA cycle is completely functional and they convert pyruvate into oxaloacetate as an anaplerotic source. Unexpectedly, inhibition of the PPP with dehydroepiandrosterone (DHEA) induces cell death only in quiescent fibroblasts (albeit to a limited extent), representing a unique example of a drug that selectively targets non-proliferating cells. Thus, despite being non-proliferative, contact-inhibited fibroblasts have a very active PPP. Lemons et al. suggest three potential explanations for their findings: high levels of glucose uptake could be required to replace potentially damaged macromolecules, NADPH produced by the PPP could be used to generate GSH and thus confer protection from free radicals and, lastly, fibroblasts require high glucose levels due to their high energy consuming function, i.e., synthesizing and secreting large amounts of extracellular matrix components. Importantly, contact-inhibited fibroblasts are not a unique example of quiescent cells with high level of glucose uptake. Hematopoietic stem cells (HSCs) also prefer glycolysis to oxidative phosphorylation in a metabolic pattern driven by HIF1α, but not just consequently to the hypoxic niche were they are found (13). Moreover HSCs’ metabolism (where mitochondrial activity is inhibited) is important to keep the quiescent status of these cells (14).

## Aerobic Glycolysis: Regulation of Protein Translation

So far, we have described examples where aerobic glycolysis is not linked to proliferation. However, proliferating cells normally switch their metabolism to aerobic glycolysis for all the reasons we mentioned in the previous paragraph. In this section, we discuss an example where aerobic glycolysis accompanies cellular proliferation but is required for translation of signaling proteins, and not for proliferation itself. A recent study of the metabolic changes accompanying T cell activation (when they undergo a highly proliferative phase) revealed that T cells not only increase their glycolysis rate, but they also increase their oxidative phosphorylation in comparison to naïve T cells (15). Moreover, in the initial 48 h of activation, T cells actually rely more on their mitochondria than on glycolysis, they survive and proliferate when fed with galactose (which is not a substrate of glycolysis) instead of glucose, and their proliferation and activation is extremely sensitive to mitochondrial inhibitors. Thus at a first glance, T cell activation seems to be independent of glycolysis, so why is there an increased glycolytic rate in this phase? In this respect, a detailed analysis of the full functional spectrum of activated T cells, including cytokine release, revealed some interesting novel observations. T cells fed with galactose instead of glucose secrete much less interferon γ (IFN-γ) and interleukin 2 (IL-2) compared to their glycolytic counterparts. The mechanism proposed to explain this phenotype is that a key metabolic enzyme – glyceraldehyde 3-phosphate dehydrogenase (GAPDH) – can also act as an mRNA binding protein. When glycolysis is inhibited (e.g., in galactose) the enzyme is not engaged in its main function of converting glyceraldehyde 3-phosphate to d-glycerate 1,3-bisphosphate, but rather binds to the 3′-UTR of the IFN-γ mRNA, thereby preventing its translation. These findings represent an intriguing example where aerobic glycolysis is not required for proliferation, but can actually regulate the synthesis of a cellular and extracellular signaling protein.

## Metabolites Can be Used as Signaling Molecules

In this last section, we would like to underline that metabolites (either directly or indirectly linked to aerobic glycolysis) can also function as signaling molecules, reinforcing the idea that metabolism is part of the cellular signaling network. For example, succinate is used by bone marrow derived macrophages (BMDM) upon LPS stimulation to induce secretion of IL-1β (16). Indeed, as recently shown, in BMDM an increase in succinate concentration creates a condition called “pseudohypoxia,” by repressing prolyl-4-hyrdoxylase (PHD) activity by product inhibition, leading to the prevention of HIF1α degradation. In turn, HIF1α can bind to the promoter of IL-1β gene inducing its expression. Importantly, secretion of different cytokines by BMDM seems to be controlled by different mechanisms since, for example, TNFα and IL-6 secretion is not dependent on succinate-mediated signaling. These data suggest that different regulatory mechanisms have evolved to control the secretion of cytokines by macrophages to ensure a specific response in different extracellular environments, such as the presence of different nutrients. Finally, while succinate functions as an intracellular signaling molecule, it appears that metabolites can also act as extracellular messengers as observed in the case of lactic acid. The Medzhitov group has recently described that lactic acid released by tumor cells induces the conversion of macrophages to tumor-associated macrophages (TAM), which show characteristics of M2 macrophages, via a HIF1α−mediated mechanism (17).

In conclusion, these are very exciting days for cellular metabolism, with old paradigms being reconsidered and different fields (such as cancer cell metabolism and immunometabolism) contributing to unravel the essential role of this complex network of reactions in cellular biology.

## Conflict of Interest Statement

The authors declare that the research was conducted in the absence of any commercial or financial relationships that could be construed as a potential conflict of interest.
